# Gouldian finches are followers with black-headed females taking the lead

**DOI:** 10.1371/journal.pone.0214531

**Published:** 2019-04-03

**Authors:** Andrias O. O’Reilly, Gerhard Hofmann, Claudia Mettke-Hofmann

**Affiliations:** 1 School of Natural Sciences & Psychology, Liverpool John Moores University, James Parsons building, Liverpool, United Kingdom; 2 Independent Researcher, Moreton, Wirral, United Kingdom; University of Tulsa, UNITED STATES

## Abstract

Colour polymorphism is a widespread phenomenon and often encompasses different behavioural traits and strategies. More recently, it has been shown that morphs can also signal consistent individual differences (personality). An example are Gouldian finches that show discrete head colour morphs in the same population with red-headed birds being more aggressive but less risk-taking and explorative than black-headed birds in the lab. The current study aimed to investigate the link between head colour and behavioural traits in a naturally risky situation in the wild by recording the order of descent at waterholes in relation to hypotheses considering conspicuousness, dominance relationships and experience. Other bird species at the waterholes were also included in the study. Adult Gouldian finches generally preceded juveniles and among the adults the least conspicuous black-headed females descended first to the waterhole. Overall, females descended before the males though this pattern disappeared later in the season likely due to family groups breaking up and releasing males from attending to the juveniles. Finally, Gouldian finches almost always followed other species, particularly Long-tailed finches, to the ground rather than taking the lead. A two-level process of decision-making seems to explain the responses best: on the first level, experience separates adults from juveniles with adults preceding juveniles and on the second level, conspicuousness acts as a factor among the adults with the least conspicuous category taking the lead. Future studies should directly test the link between head colour and personality in the wild, look more into seasonal effects and investigate whether Gouldian finches use Long-tailed finches as an indicator of safety.

## Introduction

Natural selection favours traits that give the bearer an advantage that results in higher reproductive success and over time increases the frequency of alleles coding for these traits in the gene pool at the cost of alleles of other traits. This often results in one optimal trait. However, there are also cases where different variants of the same trait exist in the same population (polymorphism) which is difficult to explain from an evolutionary point of view as the variants have to be of equal fitness to persist over time [1]. Examples are colour polymorphism where individuals differ in their appearance [2] or personality which describes consistent individual differences in behaviour [3, 4]. Mechanisms to maintain these polymorphisms include negative frequency-dependent selection [4], spatio-temporal variation in the environment [5] or trade-offs between traits [6].

It is long known that colours have a signalling function in colour polymorphic species. An example are ruffs (*Philomachus pugnax*) where males signal their mating strategy with their plumage colour. Black-brown birds are territory holders, birds with a white ruff are satellites and hang around male territories and female-like sneakers try to get copulations under disguise [7]. Likewise, the colour red often signals aggression and results in conflict avoidance as other individuals are less likely to approach [8]. More recently, it has been shown that colour polymorphic species can also signal their personality. In melanin-based colour polymorphism, such as in the barn own (*Tyto alba*), this is due to pleiotropic effects of genes affecting not only plumage coloration but also behaviours and physiology [9]. For example, darker morphs are more aggressive, explore more and are bolder [10, 11]. The only example for a non-melanin-based colour polymorphism comes from the Gouldian finch (*Erythrura goldiae*). Here red-headed birds are more aggressive but less explorative and risk-taking than black-headed birds [12]. It has been argued that red-headed birds are more conspicuous; being more cautious reduces their exposure and risk of predation, while their aggression secures them their share of resources once safe [12].

Signalling of personality can be particularly advantageous in larger groups of varying individual composition as it reduces the need to assess behaviours of other individuals. Moreover, it can signal specific roles in a group such as leader-follower relationships as shown in rock sparrows (*Petronica petronica*) [13]. Sparrows with larger badges were more likely leaders than sparrows with smaller badges. Finally, signalling of personality can facilitate group formation which can have a strong effect on group effectiveness. For example, there is increasing evidence that living in groups of mixed personality affects individual foraging success, improve coordination and increase fitness and survival [14–18]. Irrespective of personality, leadership has been shown to be linked to specific current states (e.g. reproductive state in zebras (*Equus burchellii*) [19], experience/age e.g. in elephants (*Loxodonta Africana*) [20] and social dominance e.g. in Chacma baboons (*Papio ursinus*) [21].

Gouldian finches are colour polymorphic in both sexes with 70% black-headed birds, 30% red-headed birds and less than 1% yellow-headed birds in the same population [22]. Studies in captivity have shown that red-headed birds are more prone to stress in social situations [23] and are more aggressive than black-headed birds [8, 24], though the latter does not necessarily result in social dominance [25]. Moreover, Gouldian finches signal their personality with their head colour [12, 25] and, when tested in groups, show social conformity. Individuals adapt their behavioural response to the personality of the partner; fast exploring/ high risk-taking birds become slower/ less risk-taking with a slow partner and slow exploring/ low risk-taking birds become faster/ more risk-taking with a fast partner [26]. The only exception were black-headed birds when paired with a red-headed partner. They remained fast/ risk-taking irrespective of their partner indicating that black-headed birds can act as producers or leaders in unfamiliar situations [26].

Whether head colour morphs of Gouldian finches also differ in their exploratory and risk-taking behaviour and therefore possibly in their personality in the wild is currently unknown. However, to understand the ecological significance of this relationship an important next step is to investigate it in the wild. This is particularly important as all captive birds are derived from imports prior to 1960, when exports were banned from Australia [27].

The Gouldian finch inhabits tropical savannah grassland in Northern Australia. As food and habitat specialists, they have suffered considerable declines with only about 2,500 birds remaining in the wild [28]. They are listed as endangered by the Australian Government [28] and as near threatened in the IUCN Red List [29]. Earlier studies in the wild have shown that red-headed birds are more likely to occupy high-quality nest cavities than black-headed birds [30]. Moreover, sympatric occurring Long-tailed grassfinches (*Phoephila acuticauda*) usually outcompete Gouldian finches at nest cavities [31]. No research is currently available about how head colour morphs may be linked to personality in the wild or to which extent head colour morphs differ in their behaviour.

The aim of the current study was to investigate whether findings from the lab are applicable under natural settings in the wild. Specifically, black-headed birds were found to be more risk-taking in the lab than red-headed birds which was linked to their lower conspicuousness and aggression [12]. Risk-taking in the lab was tested by raising a predator silhouette in front of a feeder and measuring the time to return to the feeder.

Here we investigate whether black-headed birds are more risk-taking at waterholes than red-headed birds. Birds at waterholes are often prone to predation by raptors [32]. Descending first is risky as the bird is more exposed, easier to target and may also have a lower chance of escape from the ground than a bird in a tree. However, the situation may slightly differ from the feeder experiment as reasonable sized waterholes offer ample opportunity to land, though specific spots may be favoured over others due to visibility and escape routes. Birds may compete for these favoured spots similar to competition and monopolisation of other high-quality resources [33]. Which bird goes first can be linked to leader-follower relationships which can be affected by different factors (see above). Considering all factors, the following scenarios are possible. 1) Effect of conspicuousness: In parrots, the less conspicuous sex explored novel objects earlier than the more conspicuous sex [34] and in Gouldian finches the less conspicuous black-headed morph was more risk-taking than the more conspicuous red-headed morph [12]. Their lower conspicuousness may out-weight costs of more exposure to predators. Therefore, black-headed birds may descend first to the waterhole (prediction 1A). Moreover, as the females are less colourful than the males, females may precede males (prediction 1B). Data collection for the current study took place at the end of the breeding season with many inconspicuous juveniles (uniform grey-green plumage) present. Consequently, juveniles may precede adults (prediction 1C). 2) Effect of dominance: The literature is inconsistent about the effect of dominance on taking risk or taking the lead with several studies showing that subordinate individuals take greater risk to secure resources [35, 36], whereas other studies found the dominant individual taking the lead [37]. Following results from the lab [12] the less aggressive black-headed birds should descend first to secure a favourable spot, whereby red-headed birds may wait and use their higher aggression to replace the black-headed birds from the favoured spot (prediction 2A).. Similarly, as juveniles are often subordinate to adults they may drop down to the waterhole first (prediction 2B). 3) Effect of experience: More experienced birds have been shown to be less fearful [25, 38]. Therefore, adult birds may precede juvenile birds (prediction 3). This prediction contrasts with the ones before. 4) Effect of physical state: Higher demand for some resources e.g. during lactation can result in leadership [19]. Therefore, thirstier birds may take the lead. This prediction cannot be tested here though some anecdotal evidence supports this. Finally, Gouldian finches often assemble in mixed species flocks at waterholes [32] which may be an antipredator strategy. We investigated whether Gouldian finches compete with other species, particularly Long-tailed finches, to get access to waterholes (particularly favourable spots) as competition has been shown for nest cavities [31]. This adds an interspecific component to the predictions about conspicuousness and dominance. The current study only looked for a link between head colour and behaviour as birds could not be individually identified and recognised across visits to waterholes. Investigating potential relationships between individual personality and traits within morphs was therefore not possible.

## Material and methods

### Study species and location

Western Australia is a stronghold for the Gouldian finch, where it prefers western-facing slopes with open eucalyptus woodland, annual spear grass (*Sorghum* spp.) and perennial grasses [39]. It is an obligate cavity breeder preferring sites with higher densities of cavities [40]. Feeding occurs on or nearby the breeding site [41]. The presence of water close to the breeding site is essential [39]; Gouldian finches drink only once daily, early in the morning [32].

The study sites were located in two different regions in Western Australia; five waterholes were in the Kimberley region around Wyndham (15°29'08.3"S 128°07'14.9"E) and two in the Lake Argyle region (16°05'55.4"S 128°42'17.1"E). Population sizes are estimated at 50–100 adult individuals in both regions, each [42]. Gouldian finches were observed over a four-week period at the end of their breeding season in June 2017 at waterholes within or close to their breeding sites. Mean distances between waterholes in the Kimberley region were 9 km (min 1 km, max 29 km; Table 1) and visited by different populations except for two waterholes along the King River Road [30]. The two waterholes in the Lake Argyle region were 4 km apart; it is unknown whether the same birds visited both locations although waterholes were separated by a ridge.

**Table 1:** Waterhole locations and sampling frequencies.

Location	Region	Distance (km) to location 1 Camp ground)	Number of observations	Events/day	Dimensions (m)
Wyndham Camp ground	Kimberley	0	3	16; 52; 43	10 x 10
King River Road 1	Kimberley	14	2	61; 52	10 x 3
King River Rd 2	Kimberley	15	3	31; 50; 48	20 x 1
Victoria Hwy	Kimberley	28	2	16; 26	10 x 3
Pictorella	Kimberley	57	3	37; 45; 16	6 x 4
Lake Argyle 1	Lake Argyle	171	1	7	7 x 3
Lake Argyle 2	Lake Argyle	175	2	12; 18	4 x 2

Events/day: entries represent events on different days

Habitats at all sites were characterised by annual sorghum grass (*Sorghum spp*.) and a mixture of eucalyptus (*Eucalyptus spp*.) and bloodwood (*Corymbia spp*.) trees characteristic for the species [43]. The campground in Wyndham differed somewhat in that one side had annual sorghum but the other sides were open campground with eucalyptus trees. Birds visiting this waterhole came from the nearby hills (Bastian population). Waterholes were usually remains of creeks ranging in size from 20 meter length by 1 meter width to 10 m x 10 m ponds with tributaries (camp ground) to 4 m x 2 m diameter (Lake Argyle; Table 1). Waterholes were selected based on long-term use by Gouldian finches (pers. comms. Gary Fitt, Mike Fidler).

### Data collection and analysis

Data collection occurred from 6:00 am– 10:00 am each morning at a particular waterhole. Birds could not be individually distinguished and only frequencies of different classes (see below) were recorded. However, as Gouldian finches only drink once in the morning [32] we assume we did not sample the same birds twice on a given day. Most waterholes were sampled at least twice with the exception of Lake Argyle 1 as this location was only frequented once as birds moved further down due to high predation at the waterhole (Table 1). As soon as Gouldian finches landed in nearby trees, the Dictaphone was started and head colour (only adults), sex and age (juvenile or adult) of the finches present were recorded. Gouldian finches are sexually dimorphic with males having a more intensely coloured purple breast band and yellow belly than females [44]. Young of the year are uniformly coloured green/grey without showing colour polymorphism in the juvenile plumage [44]. The presence of other Estrildid finches was also noted. Irrespective of the species, the order of birds landing on the ground (noting head colour, sex and age for Gouldian finches) was identified.

We extracted frequencies of four variables: 1) Head colour of the first Gouldian finch on the ground to test whether the less conspicuous and less aggressive black-headed birds descent first (predictions 1A and 2A). We distinguished red-headed, black-headed and unknown (juveniles). 2) Sex of the adult Gouldian finch going down first (see prediction 1B). 3) Age class (juvenile—adult) of the first bird on the ground to test for any age differences (experience) in risk-taking (predictions 2B and 3). 4) First species on the ground (Gouldian finch, Long-tailed finch or ‘other’ species) to test whether Gouldian finches are more likely to follow other species than to take the lead. ‘Other’ species included double-barred finch (*Taeniopygia bichenovii*), star finch (*Neochmia ruficauda*), mask finch (*Poephila personata*), Crimson finch (*Neochmia phaeton*), zebra finch (*Taeniopygia guttata*), pictorella manikin (*Heteromunia pectoralis*), diamond dove (*Geopelia cuneata*), crested pigeon (*Ocyphaps lophotes*), magpie-lark (*Grallina cyanoleuca*), miners and honeyeaters (both Meliphagidae). We combined these species in one category as Gouldian finches seemed to follow primarily Long-tailed finches, thus relatively few data were available for the other species.

We tallied events when Gouldian finches were present in trees or on the ground. An event started when the first bird of any species landed on the ground and finished when no bird was left on the ground. We observed 530 events with Gouldian finches present, of which 394 included Gouldian finches descending to the ground, the primary data for our analyses. We pooled data across all waterhole sites except where specified.

For analyses investigating effects of head colour, sex and age on descending first in the Gouldian finch (variables 1–3) we calculated the proportion of each head colour within each sex, and the proportion of juveniles, from all recorded Gouldian finches across all sites, as morphs occur in different frequencies[22]. We used these as expected probabilities with 0.27 black-headed males, 0.26 black-headed females, 0.08 red-headed males, 0.03 red-headed females and 0.36 juveniles. The three dependent variables (1–3) were analysed together, as presence of one Gouldian on the ground can affect reactions of the others. For example, a young Gouldian on the ground may affect reactions of red-headed and black-headed birds differently. The observed frequencies of descending first to the ground were then tested against the expected probabilities with a Goodness of Fit chi-square test. Where significance was encountered, planned pairwise comparisons (chi-square) were conducted as posthoc tests. To account for any autocorrelation due to sampling the same birds at a given waterhole on different days we run the same analyses on a daily basis. In some cases we were certain that only one head colour was present. Therefore, we did a second analysis only including cases where both head colours were present (81 events with 548 birds out of 394 events with 1486 birds) to avoid any biases. Expected probabilities for these cases were 0.27 black-headed males, 0.23 black-headed females, 0.17 red-headed males, 0.06 red-headed females and 0.27 juveniles. Sex differences were further investigated by splitting the observation period into an early (sites 1–4) and late season (sites 5–7) to account for increasing independence of the young. As males usually attend to the young after fledging [44] this can affect their decision when to go to the ground. Expected values were 0.46 for females and 0.54 for males.

To test for species differences in probabilities of being first on the ground (variable 4), we compared the observed numbers of Long-tailed finches, ‘other’ species and Gouldian finches against their expected probability (1/3 each) as all three categories were usually present and exact frequencies of long-tailed finches and other species were not available. This represents a conservative approach. Sample sizes were again 394 events for this analysis. To further investigate whether Gouldian finches were more likely to follow Long-tailed finches than ‘other’ species, we created contingency tables of first species vs following species using contingency chi-square tests. Values in cells were compared with z-statistics and adjusted with the Bonferroni method. The data set used were the 530 events with Gouldian finches present (but not necessarily descending to the ground). However, as all events with only one individual drinking had to be excluded for this analysis, the sample size was 405 events. All statistical analyses were conducted in SPSS 18.

### Ethics

All applicable international, national, and/or institutional guidelines for the care and use of animals were followed. Ethical approval including sampling procedures for all locations was given by Macquarie University, Sydney, NSW, Australia (animal ethics number ARA2017-027). Permissions to work on private land was given by all owners.

## Results

**Fig 1:**
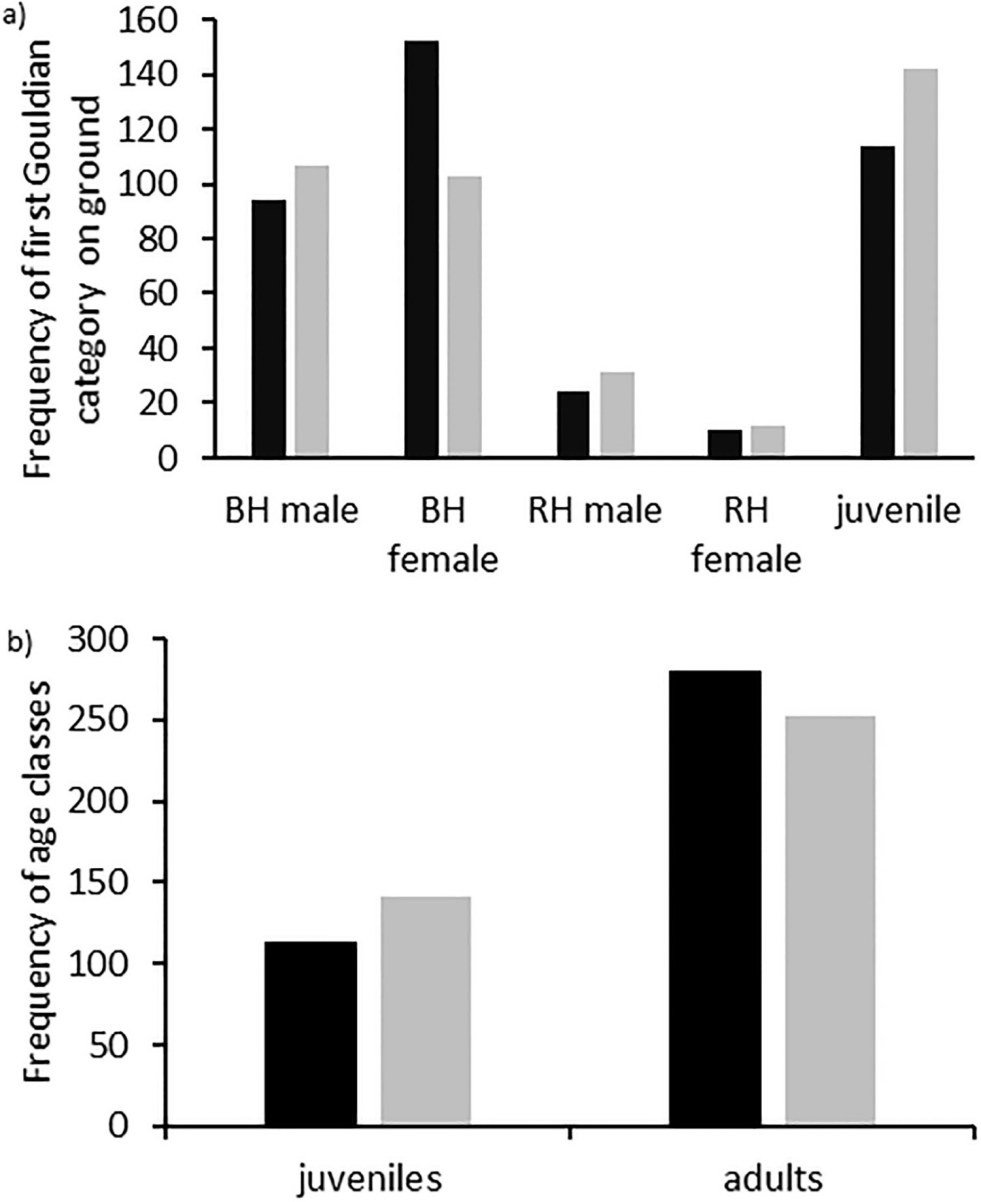
Frequency of age and sex classes and head colour morphs in Gouldian finches descending first to the ground. (A) Observed values (black) are plotted beside the expected value (grey) for each class for black-headed (BH) males and females, red-headed (RH) males and females and uniform juveniles; (B) observed and expected frequencies for adults and juveniles only (pooled across sexes and head colour morphs).

Ages, sexes and head colour morphs of Gouldian finches differed in their likelihood to descend first (Goodness of Fit chi-square n = 394, df = 4, chi^2^ = 32.956, p<0.001; Fig 1A). Black-headed females were more likely to land on the ground before all other Gouldian finches. Pairwise comparisons of age classes showed that given their presence, adults were more likely to land first than juveniles (n = 394, df = 1, chi^2^ = 8.538, p = 0.003; Fig 1B).

**Fig 2:**
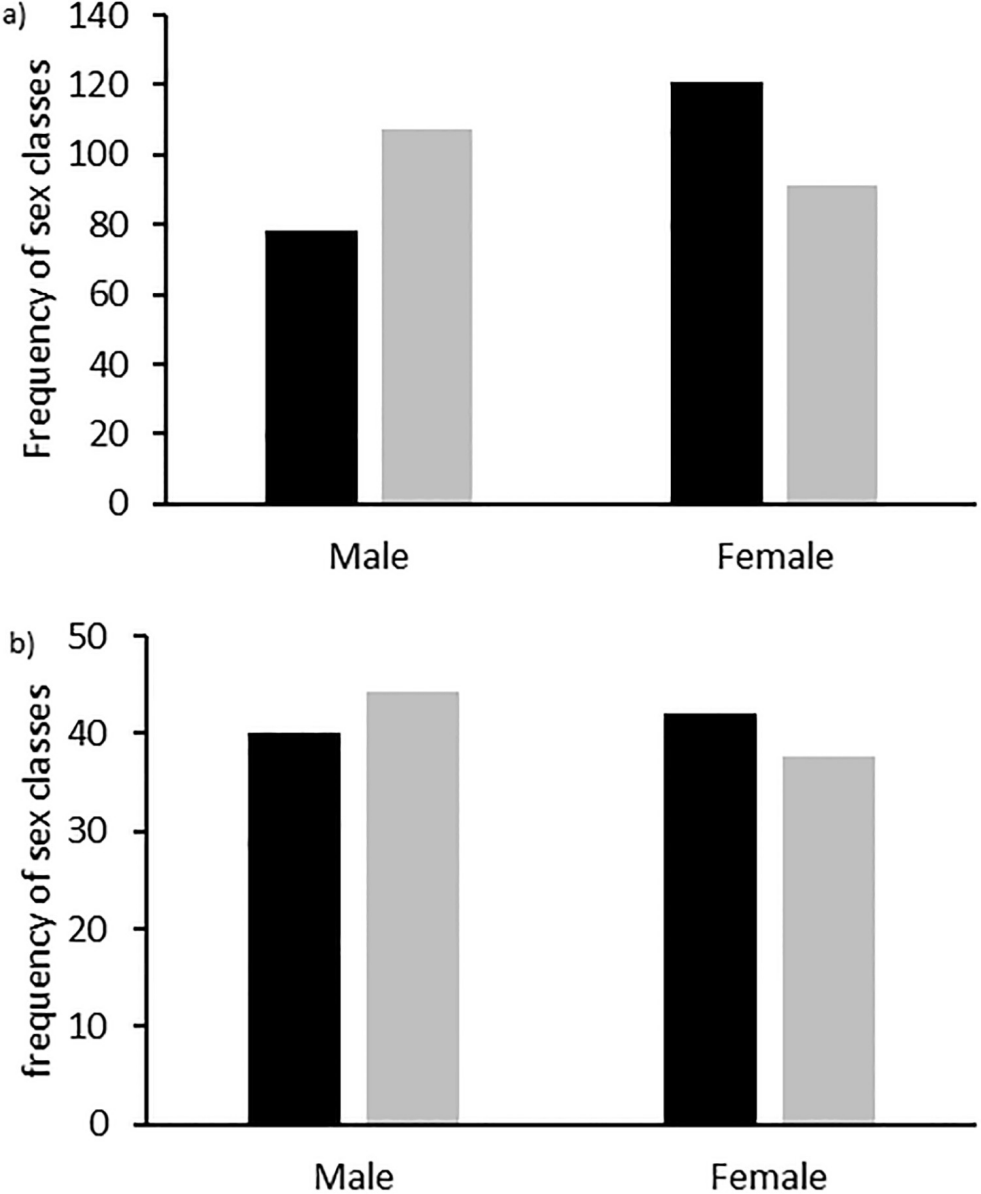
Frequencies of sex classes landing first on the ground early and late in the season. Observed (black) and expected (grey) frequencies of males and females being first on the ground early (A) and late (B) in the season.

Across all sites, females were more likely than males to descent first (adults only n = 281; df = 1, chi^2^ = 16.309, p<0.001), but this was strongest earlier in the season (early—sites 1–4: n = 199, df = 1, chi^2^ = 17.557, p<0.001; late—sites 5–7: n = 82, df = 1, chi^2^ = 0.899, p = 0.343; Fig 2)) with a trend towards black-headed females going down first later in the season (n = 82, df = 3, chi^2^ = 7.572, p = 0.056). When only considering cases where both head colours were definitely known to be present, analyses produced similar results with respect to age, sex and head colour morphs differing in their likelihood to descend first (n = 81, df = 4, chi^2^ = 13.668, p = 0.008). Restricting analyses to one particular day to account for repeated sampling resulted in the same significant differences for two days (day 1: Goodness of Fit chi-square: n = 142, df = 4, chi^2^ = 25.261, p<0.001; day 3: n = 71, df = 4, chi^2^ = 13.00, p = 0.011) and a similar but not significant picture for one of the days (day 2: n = 181, df = 4, chi^2^ = 0.165).

**Fig 3:**
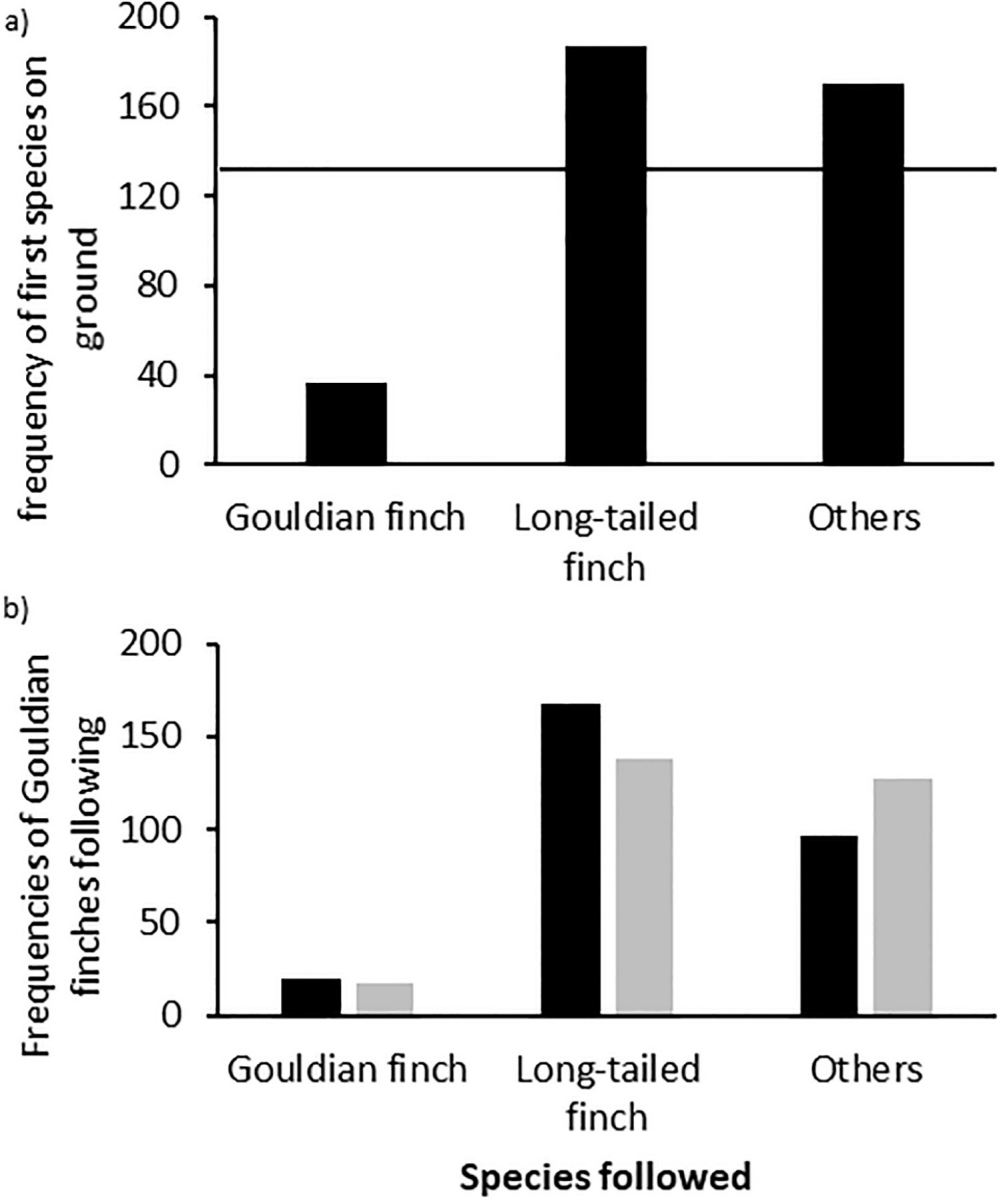
Frequency of first species on ground at waterholes. (A) Frequencies for Gouldian finches, Long-tailed finches and ‘other’ species (see text) descending first to the ground tested against an equal expectation of 1/3 for each group (black line); (B) Observed (black) and expected (grey) frequencies of Gouldian finches following another Gouldian finch, a Long-tailed finch or an ‘other’ species to the ground.

Finally, Gouldian finches were rarely the first on the ground but usually followed other species (Goodnes-of Fit Chi-square n = 394, df = 2, chi^2^ = 102.736, p<0.001; Fig 3A). Specifically, Gouldian finches were equally likely to follow other Gouldians and Long-tailed finches but avoided following ‘other’ species to the ground (Contingency Chi-square test: n = 405, df = 2, Pearson’s chi^2^ = 48.126, p<0.001; Fig 3B).

## Discussion

With Gouldian finches, the first bird descending to the waterhole were biased towards black-headed females and females in general were more likely to come down before the males. However, the sex difference was stronger earlier during the season. Juveniles were among the last to come down. In comparison to other species, Gouldian finches were rarely the first birds to descend but usually followed others—particularly Long-tailed finches—to the ground.

Considering the different hypotheses in how far conspicuousness, dominance and experience may affect a Gouldian finch’s decision to land on the ground first, only experience (hypothesis 3) got fully confirmed with adult birds generally preceding juveniles. The lack of experience in young birds may expose them to higher predation. Staying behind and following more experienced adult birds can be seen as a predator avoidance strategy by the juveniles. The result is in concordance with findings in the lab which showed that the younger bird in a pair hesitated longer to feed beside an unfamiliar object [25]. The object increased uncertainty and risk in the environment resulting in more cautious behaviour of the less experienced bird. Similar results were found in Rufous-tailed jacamars (*Galbula ruficauda*); young birds hesitated longer to attack a butterfly with novel wing patterns than adults, indicating more cautious behaviour in the former [38].

Among the adult Gouldian finches, black-headed females were more likely than other Gouldian finches to be first on the ground. This is consistent with hypothesis 1B. Black-headed females are the least conspicuous of all adults and may experience a lower predation risk than red-headed birds and the generally more colourful males [44]. Laboratory studies had found black-headed birds to take greater risk and also explore unfamiliar situations earlier than red-headed birds, irrespective of sex [12, 25]. However, sexes were never tested together in the lab. Future laboratory studies should investigate whether black-headed females are more risk-prone than any other group in Gouldian finches. The results are in line with a study investigating exploration in ten parrot species. It was found that the less conspicuous partner in a species approached and investigated changes in its familiar environment (e.g. novel objects) earlier than the more colourful partner [34] further supporting the hypothesis that conspicuousness plays a role in decision-making when to approach unfamiliar or dangerous situations. Another explanation is linked to the finding that females as a whole were more likely to be first on the ground than males though this difference weakened later in the season. At the start of the data collection many birds were still in family groups. Males primarily care for the young when fledged [44] and may have stayed with the young in the tree resulting in females being first. Later in the season family groups had split up and often pairs and young came in different flocks. This was when sex differences disappeared. Attending to the young may explain why black-headed males were not more likely than expected by chance to be the first birds on the ground; they stayed with the young in the trees surrounding the waterholes. Interestingly, Pryke and Griffith [45] found comparable investment in parental care among males of both head colours under low competitive conditions, but a stark decline of parental care in red-headed males under high competitive conditions (high proportion of red-headed males). The ratio between head colours we observed in the wild seems to be more at the lower competitive end (27% black-headed males vs 8% red-headed males; [22]) and is unlikely to have affected parental care differently. The seasonal effect needs further investigation as sample sizes in the latter part of the season were relatively low and a site effect cannot be excluded, since the more westerly located sites were sampled earlier in the season than the more easterly sites. More systematic research is required here. Hypothesis 1C was not confirmed as juveniles rarely descended before the adults.

Hypotheses about effects of dominance on decisions about when to drop to the ground were not confirmed. Juveniles as the most subordinate class did not descend first, rejecting hypothesis 2B. Among the adults, only black-headed females, but not males, descended first, supporting hypothesis 2A in part. Competition may not play a big role in this context as there was always enough space available to land and drink. A study measuring dominance in captive Gouldian finches did not find a relationship between dominance and head colour under low competition [25]. In contrast, such a relationship emerged under high competitive conditions (8, 24). While in the wild birds may compete for the most favourable spot to drink, this may not be a major driving force for decision-making.

The emerging picture of factors influencing the decision to go to the ground seems to be a two-level process. The first level is driven by experience with more experienced adult birds preceding inexperienced juveniles. The second level separates adult birds according to their conspicuousness with the least conspicuous black-headed females having the lead. These two factors accurately explain the observed pattern of descending to the ground.

Finally, Gouldian finches predominantly waited for other species to fly down to the ground before descending themselves. This is an interesting discovery. Often Gouldian finches would wait for more than 20 minutes when no other birds were around to follow others immediately when they finally arrived and descended to the ground. Such behaviour was never observed with the other species at the waterholes. The hesitancy to descend first may be linked to the colourful appearance of the Gouldian finch, which may expose it more to predation than other species. Waiting for others either to see whether the area is safe or to find safety in numbers is a known predator avoidance strategy [32]. However, Gouldian finches primarily followed Long-tailed finches. This is interesting as so far the Long-tailed finch was more seen as a superior competitor with respect to nest holes [31]. In contrast, around waterholes Long-tailed finches may act as a nuclear species [46] for Gouldian finches. Why they prefer this species above others is unknown but may be explained with their co-occurrence across the species’ range and their similar ecological requirements, although the Long-tailed finch is much more a generalist than the Gouldian finch [31]. Future research should look into this relationship in other contexts e.g. during foraging.

To summarise, the decision by Gouldian finches to descend to the ground can be explained with a two-level decision process; experience overrides all other factors on the first level with adults preceding juveniles. The second level governs decisions among the adult birds according to their conspicuousness with less conspicuous individuals preceding more conspicuous ones. Furthermore, Gouldian finches are followers at waterholes and prefer to follow Long-tailed finches more than any other species. Future studies should investigate whether the link between head colour and risk-taking behaviour is part of a personality syndrome as it has been found in controlled laboratory studies. Furthermore, future studies should look into the seasonal change in behaviour at waterholes and also investigate the possible role of Long-tailed finches as a nuclear species for the Gouldian finch in other contexts e.g. foraging.

## Supporting information

S1 Dataset Data for the first bird on the ground with respect to head colour, age, sex and the combination of sex and head colour in Gouldian finches. Data are separated by region, season, site and day.
(XLSX)

S2 Dataset Data about which species followed which one when descending to the waterhole. The first species on the ground is depicted in the first column with the species following listed in the next three columns. Species are distinguished into Gouldian finches, Long-tailed finches and ‘others’ (explanation see text).
(XLSX)
